# Iranian superwomen's career experiences: a qualitative study

**DOI:** 10.1186/s12905-021-01369-3

**Published:** 2021-05-31

**Authors:** Maryam Nosrati Beigzadeh, Hossein Ghamari Givi, Ali Rezaei Sharif, Ali Sheykholeslami, Leila Reisy, Hadi Hassankhani

**Affiliations:** 1 University of Mohaghegh Ardabili, Ardabil, Iran; 2 Department of Counseling, Faculty of Education and Psychology, University of Mohaghegh Ardabili, Ardabil, Iran; 3 Department of Midwifery, School of Nursing and Midwifery, Ardabil University of Medical Science, Ardabil, Iran; 4 Department of Nursing, Nursing and Midwifery Faculty, Tabriz University of Medical Sciences, Tabriz, Iran

**Keywords:** Iranian superwomen's, Career experiences, Qualitative study

## Abstract

**Background:**

Superwoman refers to the identity of a woman who performs several important roles simultaneously and full-time, such as being a wife, mother, and homemaker while holding a job. This study aims to examine the career experiences of Iranian superwomen who maintained their mental health while holding multiple roles.

**Methods:**

Data for this qualitative study were collected via semi-structured interview and analyzed using conventional content analysis. The study participants were 12 multi-role women from different occupations in Tabriz, North West of Iran selected through purposive and theoretical sampling. The women’s mental health status was assessed using Mental Health Continuum-Short Form questionnaire before participating in the research**.**

**Results:**

The results were classified into three main categories. The first category included Underlying Factors of Job Experience with six subcategories, including Limited job opportunities for women, Educational context (mindset shaped in the parental home), Commitment or freedom in obtaining a job and its continuation, Personality traits, and Non-cognitive skills (emotional intelligence and spiritual intelligence); the second category included the adopted strategies to solve career problems with two subcategories: effective strategies, including the benefits of having a mindset of abundance and having a vision and strategic planning, and ineffective strategies, including the benefits of having a mindset of scarcity; and the third category included Perceived Consequences of employment with two subcategories: psychological consequences and social consequences, with both subcategories including some positive and negative further subcategories. Moreover, a conceptual relationship between the main categories and the subcategories was evident.

**Conclusions:**

The content obtained not only supports the findings about the experiences of multi-role women in cultures outside Iran, but also points to the unique aspects of Iranian superwomen's life experiences and narratives. The findings of this study can help us perceive the real career experiences from the perspective of Iranian professional women with multiple roles.

**Supplementary Information:**

The online version contains supplementary material available at 10.1186/s12905-021-01369-3.

## Background

Each individual's life experience is influenced by social and cultural beliefs, values, and unique life events. Life Experiences and challenges are a facilitating factor for mental development and psychological resources have a significant impact on how people evaluate the challenges of their lives, how they cope with them, how they integrate them into their life story and ultimately create unique experiences [1] Research has shown that individuals' reflective narratives are critical to how they perceive important life events [2].

Several researchers in the field of occupational therapy also use narratives to examine people's life and work experiences [3, 4]. In general, research on life-work experiences has expanded over the past five decades due to the tendency to change the nature of gender roles, family structure, and employment [5] Researchers have been studying the problems faced by mothers in the workforce for decades [6, 7]. The idea of "participating in multiple roles simultaneously" or "doing it all together" in which women play a leading role has become a valuable social norm [8]. In the West, women's participation in multiple roles has been considered a key feature of their identity [9]. In Iran, on the other hand, there is still a dual perception of maternal employment, especially in certain professions. Iranian families are less likely to employ women in full-time or managerial jobs. The largest proportion of women's participation in the public sector belongs to the Ministries of Education (69.7%) and Ministry of Health, Treatment and Medical Training (14/43%), which shows the focus of women in the two professions of teaching and nursing, which are the continuation of the same female domestic jobs. Meanwhile, the proportion of women in the other ministries ranges from 0.02 to 4.9% [10]. "Superwoman" is a metaphor that refers to the identity of a woman who performs multiple important roles within marriage, motherhood, employment, and household responsibilities simultaneously and full-time [11]. In academic literature, "superwoman" is often described as a woman who holds multiple roles and is responsible for both employment and household responsibilities full-time [12]. In Woods-Giscombé's research, a superwoman is a social phenomenon that has influenced the way African-American women experience. In this study, the following specific characteristics were introduced for the superwoman model: commitment to roles, experiencing stress, commitment to suppressing certain emotions, and helping others. The results of this study showed that women with Superwoman ideology experience both advantages and disadvantages. Advantages include being able to protect themselves and their family/club and having a sense of influence. Disadvantages include relationship pressure, emotional eating, poor sleep, and excessive levels of stress (anxiety, depression, poor mental health) [11].

Another study showed that the quality of roles in relation to the multiplicity of roles has a greater impact on mental health and quality of life. In other words, having multiple roles does not necessarily have a positive or negative effect on people's experience [8]. Therefore, the applicability of traditional gender roles to current gender roles or behavior is increasingly ambiguous between women and men [13]. A review of research on women's employment and their multiple roles reveals a number of similar perspectives and also theoretical differences that appear to be rooted in cultural and social differences. Moreover, no research could be located on the experiences and perspectives of Iranian superwomen in relation to their work.

In recent years, Iranian women have had new experiences related to their traditional roles and the conflict between traditional and modern expectations. And they play another role as working women in addition to the exclusive duties of motherhood and marriage. Despite the relative expansion of women's employment, the issue of multiple roles for Iranian women is still controversial. Therefore, the researcher needs to elicit the truth from the daily experiences of the participants in order to examine the many undiscovered facts in this regard [14]. So far, there has been a plethora of studies on the impact of employment on social and family relationships, but no research has been conducted to shed light on the professional life experience of Iranian superwomen from an exploratory perspective. Since employment is an important part of a superwoman's life, the nature of these women's perception and experience of their work is expected to play a significant role in other aspects of their lives. Accordingly, this study was conducted to explore the occupational experiences of Iranian women.

## Methods

### Study Design

A qualitative method with a conventional content analysis approach was used to conduct this study. The qualitative method emphasizes deep understanding, complexity, and details of the phenomena under study, and the researcher is actively involved in the research process. In conventional content analysis, most of the data are obtained through interviews, and interviews with individuals allow us to understand the experiences and perceptions of the participants and obtain richer data from their experiences [15]. Data were collected through semi-structured interviews based on the relevant literature [16–19]. The semi-structured in-depth interviews began with an open-ended question, "Considering that you have multiple roles, how would you describe your work life?" Exploratory questions were gradually used according to the participants' dialogs to extract further details and clarify the interviewees' explanations and examples. The questions also specifically aimed to elicit information about participants' perceptions of the concept of a woman's job, the concept of a superwoman, taking up a job, the reason for continuing the job despite having several other roles, and the strategies used to maintain a balance between personal and work life. These general questions were evaluated by five experts (Additional File 1).

#### Setting and participants

Among the multi-role women living in Tabriz, northwestern Iran, 12 women who met the inclusion criteria were purposively selected. The inclusion criteria were that they had been employed for more than two consecutive years, were married with children, and were willing to participate in the study. Participants also completed the Mental Health Continuum-Short Form before taking part in the survey and could only participate if their score was above average (*p* > 50). Factor analysis revealed that the MHC-SF replicated the three-factor structure of emotional, psychological, and social well-being found in US samples. The internal reliability of the total MHC-SF scale was 0.74. The total MHC-SF score correlated 0.52 with a measure of positive affect, between 0.35 and 0.40 with measures of generalized self-efficacy and life satisfaction, and between 0.30 and 0.35 with measures of coping strategies, sense of coherence, and community collective self-efficacy. The total score of the MHC-SF correlatedat 0.22 with the total score of the General Health. Questionnaire. In Iran, Khalili Vernakshi (2015) evaluated the psychometric properties of this scale in his research and its reliability was reported with a Cronbach's coefficient of more than 0.70 [20].

#### Data collection

Data collection began after receiving the Code of Ethics from the Ethics Committee of Iran-Ardabil University from Medical Sciences (IR. ARUMS. REC. 1398.552). The interviews were conducted between June and December 2019 in Persian language. Eleven women were initially interviewed. Due to data saturation, no new data emerged. However, another interview was followed up for confirmation and accuracy. Thus, the number of participants was twelve and the demographic characteristics included: occupation, employment history, education, age, marital history, number and age of children, and the score of their mental health questionnaire, which is shown in Table 1. The interviews provided relevant and valuable information and the depth of the descriptions was consistent with the purpose of the study. Therefore, this sample size was appropriate for this study and achieved theoretical saturation. Concurrent with the information gathering, analysis was conducted using the contractual content analysis method to identify "employment experiences." Content analysis focuses on the life experiences, interpretations and meanings that people face [21]. Each interview lasted between 60 and 140 min and all interviews were conducted by the researcher who had professional experience as a counselor. The researcher also made an effort to build a good relationship with the participants in individual sessions. After agreeing on the location of the interview (partly in the clinic and partly at the participants' workplace) and in order to adhere to the ethical principles of research, the purpose of the research and confidentiality were explained and informed consent was obtained to participate in the research and consent to record the dialogs through the protocol. The informed consent obtained was written and signed by each of the participants.

**Table 1:** Demographic information of participants in the research

Code	Job	Employment history (years)	Education	Age	Marital history (years)	Number and age of children years old	Score of mental health *P* > 50
1	Founder and principal of kindergarten	9	MA	42	20	2 (11 & 18)	67
2	Founder and principal of non-profit school	4	MA	34	16	3 (2, 10 & 13)	61
3	Secretary	7 (in the current job)	MA	36	3	1 (15 months)	55
4	Psychologist	2	MA	37	22	2 (15 & 21)	60
5	Teacher	22	BA	44	22	2 (12 & 20)	70
6	Lifeguard and sport coach	16	Ph.D	35	10	1 (4)	63
7	Manager of a private company	3 (in the current job)	MA	50	30	2 (27 & 29)	61
8	Computer teacher	14	BA	38	11	1 (3)	50
9	Deputy principal	29	BA	48	27	1 (26)	64
10	Theater actor	18	BA	46	22	1 (17)	60
11	Owner of a homemade food restaurant	6	Diploma	38	20	1 (18)	60
12	Nurse	12	Ph.D. student	40	13	1 (10)	50

#### Data analysis

Data **were** analysis done manually by conventional qualitative content analysis. In this method, the analysis was conducted simultaneously with data collection. In this method, codes and classes were extracted directly and inductively from the raw data. First, immediately after each interview, all conversations (including words, tone of voice, pauses, and laughter) were transcribed and typed. In order to increase credibility, the member- checking method was used. This means that the transcripts were presented to the participants (some of whom refused to read them) for comment. To complete the interviews, it was necessary to repeat some interviews. Then, the researcher extracted the semantic units and coded them in the form of initial codes by repeatedly reading the interviews. Using the constant comparison method, the collected data were coded, compared with other data and notes, and reduced into concepts and categories. The data were categorized and subthemes were formed by comparing similarities and dissimilarities of the codes and prolonged engagement with the data to provide a description of, increase understanding of, and gain knowledge about the phenomena under study; and finally, the latent content of the subthemes was formulated into a theme." Another way to assess the credibility of qualitative research is through "triangulation techniques". This method refers to the use of multiple data sources to present findings and overcome bias. In this research, diversity was considered in the selection of participants in terms of their occupation, role responsibilities (number and age of children), social status (occupation and education) and economic status (income and housing).

To ensure transferability, a full table of participant demographics, including job title, employment history, education level, age, marital status, number of children, and mental health questionnaire results, is provided (Table 1).

For consistency or trust, all raw information (recorded conversations, field notes, and interview texts) was retained and reproduced without attribution where necessary.

Finally, to increase credibility, the method of "researcher credit" was used. This means that the interviewer (first author) had sufficient experience in interviewing, interviewing, and conducting counseling interviews and had experience in conducting a qualitative research project. Also to increase credibility, a quote that matched the language and tone of the participant was provided.

## Results

As can be seen in Table 2, the study included three main categories: underlying factors of work experience with six subcategories, strategies used to solve workplace problems with two subcategories and several subthemes, and perceived consequences of employment with two subcategories and several subthemes**.**

**Table 2:** Major categories, sub-categories and sub-themes related to Iranian women’s job experience

Main categories	Subcategories		
Contextual factors of job experience	Limited job opportunities for women		
	Educational context (mindset formed in birthparents’ home)		
	Obligation in getting a job and continuing it		
	Freedom in getting a job and continuing it		
	Personality characteristics		
	Non-cognitive abilities	Spiritual intelligence	
		Emotional intelligence	
Strategies adopted in solving job problems	Effective strategies	Benefiting from abundance mentality	Win–win thinking
			Use of environmental potentials
			Use of individual potential
		Having a vision and strategic planning	
	Ineffective strategies	Benefiting from scarcity mentality	Lack of proper use of opportunities
			Win-lose thinking
			Fear of risk
Perceived consequences of employment	Psychological consequences	Positive consequences	Independence, self-esteem, happiness…
	Social consequences	Negative consequences	Feeling of jealousy, Lack of interest in life Feelings of guilt, Feeling of anger towards others, Feeling of inadequacy
		Positive consequences	Social dignity
		Negative consequences	Pressures from multi-role expectations, Perceived gender pressures

### Underlying factors of work experience

All participants confirmed that a number of background and underlying factors contributed to the formation of their work experiences. These factors were grouped into six main categories, including limited job opportunities for women, educational context (the mindset formed in the parental home), and obligation to find and continue a job, freedom to find and continue a job, personality traits, and non-cognitive skills.

### Educational context

The prevailing culture and upbringing in childhood and adolescence influence many decisions and non-decisions in adulthood.

"… My mother was had job, and this mentality had been formed in me since childhood that a girl should be independent…" Participants 6, 9, 3, and 8.

"… My father always said that a girl should be financially independent so that her husband would not dominate her…" Participant 3.

### A commitment to getting a job and continuing in it

Choosing a job without recognizing one's talents and desires, or based on a compulsion to choose and continue in the job due to existing constraints, has a direct impact on the quality of an individual's work experience.

"… I was eighteen at the time and I just did not know what I wanted. I just wanted to be independent …" Participant 3.

"… If I had answered a few more questions on the entrance exam, I would be on a different path now …" Participant 12.

#### Freedom to get a job and continue with it

In contrast, making a conscious decision and continuing out of personal interest and desire create different experiences for people.

"… If I were to go back in time, I would choose the same path again despite all the difficulties …" Participants 9 and 6.

"… Our life does not depend on my income and I continue my job out of interest …" Participants 10, 9, 2 and 5.

#### Limited job opportunities for women

In developing countries, women have fewer employment opportunities than men and often work in the informal sector with low wages. In this study, some of the female participants believed that limited job opportunities influenced their work experience.

"…When I finished high school, I became a teacher, as is the custom for girls in our village. This job is more suitable for a woman …" Participant 5.

"… After I got my master's degree, I worked in different places, but I came to the conclusion that my field was male and men had priority in hiring … I ended up having to work in a job that had nothing to do with my education …" Participant 8.

#### Personality characteristics

Personality characteristics or personality traits are an important underlying factor in determining personal and organizational behaviors. We saw common personality traits in a number of participants that can be divided into two subcategories: Personality Strengths or positive personality traits and Personality Weaknesses or negative personality traits.

### Positive personality traits

Some of the participants in this study had several positive personality traits including:

*Persistence:* "… I have worked hard to get to my current position. I continued my education while my family and husband were in another city and I had a young child and was pregnant with my second child …" Participant 2; *Liveliness and humor:* "… All my colleagues notice when I do not go to work for a day because I try really hard to be warm and lighten up the gloomy atmosphere with jokes …" Participants 6 and 5; *Discipline:* "… My work schedules always go in a certain order so that if I can not go to work, my deputy knows what she has to do that day …" Participant 2; *Seeking novelty and exploration:* "… I remember being curious as a child …, I am the same in my job and I look for the latest topics …" Participant 5; *Optimism:* "… It's true that I do not have a high professional position and it's in the service category, but I am really satisfied that the customers leave this place with satisfaction" Participant 3; And *hope* "… the only thing that kept me going in these difficult times was my hope to achieve my long-term goals …" Participant 9.

### Negative personality traits

A distinct list of personality weaknesses expressed by participants who believed such traits could play a direct role in the work experience included:

#### Excessive use of some defense mechanisms

Denial of the problem, projection of the problem, and avoidance of problem solving were among the defense mechanisms mentioned.

*Avoidance:* "… I did not follow it. The system is flawed and it can not be solved …" Participant 8; *Projection:* "… I think that someone who was able to get what he/she wanted must have either a sorority or a rich husband …" Participant 7; *Denial*: "… I do not think about thing that nervous me!" Participant 3.

#### Lack of discipline

Lack of discipline in the sense of orderliness and conscientiousness is one of the personality factors in the development of vocational experiences: "… My life situation gets out of hand when I act at the last minute, and it has little to do with lack of time. I think it's kind of a personality trait because it's the same on off days" Participant 4.

### Self-doubt or lack of confidence in others

Some of the narratives indicate pessimism and lack of confidence in self or others: '… It has nothing to do with the behavior of my colleagues. I just cannot trust them or be very close to them …" Participant 8.

Self-doubt: "… How effective can a person be in a small work environment?" Participant 7.

### Laziness and Indolence

Laziness and indolence was another personality weakness reported in the narratives:

I am lazy to do some work. I do not even know how time passes.

### Do not want more

Do not want more or being content is another personality factor observed in people who, under the guise of being satisfied with what they have, do not put in more effort and do not set long-term goals. These people are usually not motivated to progress and improve.

"… Insurance was the only thing that mattered to me. Although my professional position has nothing to do with my education, I am satisfied …" Participant 3.

### Non-cognitive skills

Non-cognitive abilities refer to constructs beyond cognitive intelligence and learning. Non-cognitive aspects of intelligence include emotional, social, and other skills. The data in this study are based on the two categories of emotional intelligence and spiritual intelligence.

#### Emotional Intelligence

The basic components of emotional intelligence, including the ability to understand one's own emotions and those of others and to relate to others, were saturated in the statements of the participants in this study.

"… At that moment I just tried to put myself in my boss's shoes …" Participant 9.

“… I am known among my colleagues to be adaptable …” Participant 10.

#### Spiritual intelligence

Using spiritual intelligence, people define a "why" for their lives so they can make an effective "how." Concepts such as professional ethics, belief in a superior processor power, honesty, conscientiousness, and a search for meaning in work are included in the definition of spiritual intelligence. To explain, we will use some examples from the participants'narratives: "… I made a promise to perform wholeheartedly on stage to help people feel better about themselves…" Participant 10. "… My work is valuable to me because I want to untie the knot of people's problems …" Participant 4. They talk about professional conscience and values of professional ethics: "… I was repeatedly offered bribes and I needed the money but I did not take it …" Participant 1. Or they make a personal meaning for their profession: "… Maybe not from the beginning, but soon after I was hired, my job made sense to me. I was no longer so results oriented and did not think like some of my colleagues that I should work as much as my income and the process became rewarding for me …".

#### Adopted strategies in Solving Job Issues

The second major class of codes related to the strategies adopted by a superwoman in dealing with her professional problems. These strategies were divided into two subcategories: effective strategies and ineffective strategies. The subcategories of her effective strategies were divided into two subcategories: Profiting from Abundance Mentality and Creating a Job Perspective and Strategic Plan:

#### Profiting from abundance mentality

The key principle of the abundance mentality is that resources are available to all, and the more you help others, the more you are helped. In a way, the difference between successful and less successful people depends on such mentality. According to this definition, the participants' performance was divided into three subcategories:

#### Have win–win thinking

Win–win thinking is a mindset that constantly strives to create mutual interest in all aspects of communication, and it is based on the principle that everything is abundant for everyone in the universe. It also means that one person's success does not depend on another's failure.

"Being destitute does not worry me much. I believe that God blesses us. We have this view in our religion that if you wish well for others, you will have the blessing…" Participant 1.

"… He was my competitor, but when he asked me for advice, I gave him all my experience as a founder …" Participant 2.

#### Harnessing environmental potential

People who have an abundance mentality trust others and use the experiences of successful people and make the most of the least environmental potential.

“I always try to listen and participate in research projects as determined by the administration." Participant 5.

These people believe that unity is not defined by being one, but by being complementary, with one's weaknesses being compensated for by the strengths of others.

"…Even the smallest ideas I received at group meetings were taken seriously and I thought about them. After a while my staff took the meetings more seriously and we now have good collective participation in our school …" Participant 1.

"… I never thought that my idea is ineffective in the group because I am not the boss. I say what comes to my mind about the progress of the company …" Participant 3.

#### Using individual potentials and trying to acquire more skills

People with an abundance mentality are not afraid of the unknown and believe that anything is possible. They overcome their fears and focus their thoughts on what they have, not what they do not have. They do not derive their feelings of peace and security from the beliefs of others and material possessions and do not depend on external factors. Therefore, external events do not have a strong influence on their inner stability, as they rely on their own abilities. "… Some people think that if the management changes, their job situation will change a lot, but these things are not important to me. I do my job well …" Participant 9. "… Well, the situation there was not good and everyone was used to it … I finally decided to leave there. Great things happen when you stop changing your environment and focus on yourself … After that, I thought about starting a clinic and putting my energy into it … Now the situation is very different …" Participant 4.

#### Have a vision and plan strategically

The thinking style of some individuals resembles reverse engineering and a comparative perspective. Such individuals first paint a clear picture of their long-term career goals and then move on to smaller goals and daily tasks and have a clear and defined path they want to achieve. These people do not suffer from indecision or doubt when making career decisions.

"… I even have a plan for when I retire …" Participant 1. "… Since I have been in this job, I have sat down and written down where I want to go and set my goals in the form of long and medium term plans. I also plan daily based on these goals …" Participant 5.

#### Ineffective strategies in solving work problems

In the participants' narratives, we noticed the use of some ineffective strategies in solving work problems. We grouped these strategies into a category called "profiting from a scarcity mentality." This subcategory itself could be divided into three separate subthemes.

#### Profiting from a scarcity mentality

People with a scarcity mentality believe that there is only a limited amount of everything, and the more others benefit from that limited amount, the smaller their share will be. The narratives of individuals whose strategies in various work situations were influenced by this view could be divided into the following three subcategories:

#### Win-lose thinking

The fundamental belief in a work environment that evolves on the basis of scarcity mentality is that competition leads to survival of the best. According to this thinking, people view someone else's victory as their own failure and suffer greatly from the sharing of privilege, credibility of power, and the benefit of others.

"… I constantly check the number of customers of other companies and compare them with our company. The situation is disappointing. Companies are growing fast …" Participant 7.

"… Unfortunately, some of the founders of the non-profit centers do not act openly because they think that if you help, you expose yourself and your competitor will quickly take your place …" Participant 2.

#### Lack of opportunity

People with a scarcity mentality constantly look at the progress of others and try to reassure themselves with various excuses that leave no room for seeing what they themselves have. Thus, they do not take advantage of a single opportunity. An example of this is:

"… Can I think of promotion with this minimum income? Those who attend these skill courses must either have rich husbands or have no financial needs…" Participant 8.

#### Fear of risk

Win-lose thinking style leads to fear of failure and rejection because people's decisions are influenced by scarcity mentality and competitive attitude, not by personal skills. Such people are not interested in taking the risk and are more interested in safety.

"… I have not thought about promotion because it's high risk. At the moment at least I have a fixed salary and I do not want the situation to change …" Participant 3.

#### Perceived consequences

Each of the participants indicated in their narratives that employment has consequences for them. These consequences could be divided into two subcategories of positive and negative consequences, and each subcategory was then divided into two smaller subthemes labeled positive and negative social consequences and positive and negative psychological consequences.

#### Positive social consequences

In recent decades, cultural and social changes in Iran, along with increased awareness and skills, have made employment a social demand for some women. Women's employment has increased their income and consequently increased their access to social capital such as education, hygiene, recreation, and improvement in the Human Development Index and shaped their social identity.

#### Social dignity

The women in this study who were satisfied with their current situation despite the multiple challenges confirmed that they perceived more social dignity from their environment than before employment.

"… Most women look at me with admiration and ask me how I can manage all these roles, especially when I have a young child …" Participant 2 "… I feel that I have gained the absolute trust of my husband and the people around me because I have shown my abilities in several roles. They ask me for advice on their own problems and accept me as a counselor …" Participation 5.

#### Positive psychological outcomes

Feeling productive and having professional success can fulfill some of the psychological needs of humans. In addition to positive social consequences, this group of participants shared common experiences of positive psychological consequences. These consequences were categorized as positive emotions from employment.

"… This work gives me a sense of dignity and worth …" Participant 1, "… I feel independent and free in my actions …" Participant 6, 9, 2 and 1.

#### Negative social consequences

In contrast, some of the participants shared experiences of negative social consequences of being multi-role. These narratives are perceived as two sub-categories: perceived gender pressures and pressures resulting from multi-role expectations.

#### Perceived Gender Pressures

In the modern world and with the evolution of public consciousness, gender role attachment has become less important, especially for women, and factors such as women's desire for social status and their pursuit of academic specializations and professional skills are intertwined. At the same time, gender inequality and sometimes violence against women in the workplace are perceived in various forms. Here are some examples of these statements:

"… If I were not a woman, I would make more progress in my career …" Participant 11.

"Some women flirt and the boss helps them with their work. But I am self-possessed and behave in a serious way, so I always lag behind my other colleagues …" Participant 2.

"…I have worked in a few other jobs and after a while I realized that the environment was not safe at all. I mean my bosses were staring at me with googly eyes, which was really annoying and made me leave my job immediately…" Participant 3.

#### Pressure from multi-role expectations

Women are required to take on other roles in addition to their job responsibilities, such as raising children and maintaining a family network. Having multiple important roles can lead to role conflict and the resulting pressure. This conflict can be caused or exacerbated by job pressures, inflexible working hours, an increased number of working hours, and lack of support from those around them, unequal sharing of household tasks and caring for a young child or the elderly.

"… I was not an official employee, I would have lost my job if I had gone on maternity leave, so I had to go to work until the last days of my pregnancy …" Participant 3. "… I feel like I am ignoring myself in order to keep going. I ignore my needs for fun, recreation, free time, etc. …" Participant 10.

#### Negative psychological consequences

Women's dissatisfaction with their status and role has increased as they have become more involved in society. The consequences of this dissatisfaction may take the form of passive responses such as emotional distress and stress. Participants in this study reported the following:

*Feelings of jealousy* "… sometimes you get jealous because you always see your colleague's life …" Participant 8.

*Lack of interest* "… I would not have gone on if I had not had to, and I would have let go of that chronic tension …" Participant 12.

*Feelings of anger towards others* "… some of my female colleague’s have not own bank cards, which means they do not have the right to spend their own income. Such women really need to be helped mentally, and I see how badly they are doing…".

*Feelings of inadequacy* “I am always stressed and I do not feel good enough in this job…" Participant 7.

*Feelings of guilt* "… When I first put my child in kindergarten and then went to work, I hated myself and wondered, what's the point of teaching so many children when my own child is crying in kindergarten?" Participant 1.

**Fig. 1:**
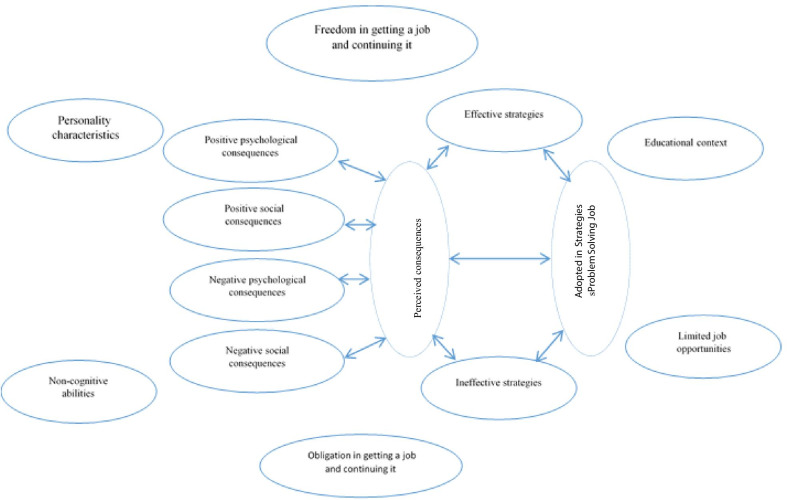
Main categories, subcategories and subthemes related to Iranian women's work experience

After a brief review of the categories obtained and rereading the interviews, a circular relationship between the categories emerged (Fig. 1). It can be concluded that based on the concepts extracted from the data, the life experiences of the multi-role professional women who participated in this study were influenced by six underlying factors that in some way affected their choice of professional problem-solving strategies. This had a reciprocal effect on the shaping of occupational experiences and the psychological and social consequences of those experiences (Fig. 1). The following examples from participant (9)'s narratives reflect this relationship:

"...As a child, my financial independence was important to my parents ... (educational context) ... Although it was very difficult, if I went back in time I would choose the same career path again ... (freedom of choice) ... Anyone who hears my life story cannot believe that a woman can show such perseverance to try to keep her job despite all obstacles ... I was very hopeful in those days and also optimistic about the future ... My food was prepared earlier than that of our neighbor who was a housewife, and I always had a certain order of doing things ... (Personality traits) ... there are moments when you would not really expect it, but you have to make a decision right away that is the best choice at that moment ... (emotional intelligence) I never thought of not paying much attention to my job because of the low salary. I had a personal purpose for all the extra activities I did ... (spiritual intelligence) ... I worked around the clock so I could make the most of the opportunities the office gave me to help raise our school's sports teams. We always won and my efforts paid off ... There were always people who wanted to throw stones, but I did my job ... (Abundance mentality) ... My son says that he is proud to have such an active mother ... My parents are proud of me too ... My husband has always talked about my abilities ... (social implications) ... It gives me a special sense of pride and self-esteem and one becomes more hopeful to go on and be sure that it was worth choosing this path ... (psychological consequences)"

## Discussion

The findings of this study can help us understand the real life work experience from the perspective of working women. The themes obtained point to the unique aspects of Iranian superwomen's life experiences and narratives and also support the findings on women's experiences of multiple roles in transnational cultures. What distinguishes the data of this study from the findings of studies on non-Iranian superwomen are the influence of the underlying contexts on shaping their professional experiences. The underlying contexts of this study included educational context, freedom or obligation to get and continue a job, personality traits, and constraints in job opportunities, and non-cognitive skills, which to some extent influenced the selection of problem-solving strategies in the workplace. On the other hand, the strategies chosen had a significant impact on positive or negative psychological and social consequences that resulted.

Participants in the present study felt that the educational context and prevailing attitudes in the home had either a positive or negative influence on the motivation and quality of their work experience. As Murdouk notes in his book, "some women find that their efforts to succeed and gain the approval of others are based on the satisfaction of their parents, especially the inner father." Jung's analytical psychology theory states that such women, who hold the title of "superwomen" but mimic the "men's championship journey" model, have only two choices; either they must compete hard in the male-dominated culture and become "successful" or they become dominated and dependent. But this theory also proposes a third way, which is to "live fully as a woman and enjoy your whole being and position in the universe" [22]. In explaining the underlying category of freedom or obligation in taking up and continuing work, it can be stated that Iranian women are not legally barred from participating in economic activities and they have full control over their economic affairs from the perspective of religion [23]; however, some factors influence their participation or non-participation in society. The present study has tried to discover these factors. In recent years, Iran has seen a sharp increase in inflation and a sharp decrease in the purchasing power of families, to which men's sole income cannot respond. Therefore, some women are forced to enter the labour market and earn money. People in forced labour pay scant attention to their professional preferences and interests. Subsequently, they make decision based on coercion and the economic conditions which they are entangled with. This finding is consistent with sociological theories of occupational choice. Among the adherents of this theory are Caplow, Miller, and Form [24, 25]. This forced employment in the absence of proper supportive institutions (such as the lack of appropriate conditions for pregnant or lactating mothers and young children, the lack of promotion of the culture of men's participation at home, etc.) has doubled the pressure of multiple roles for women, leaving them without positive experiences of their work. Recognising the importance of institutional support, the latest findings (2019) show that we are now seeing competition in the provision of extended paternity leave and other "family-friendly" benefits to attract and retain young people in companies that rely on young talent [5]. Given flowchart #1, superwomen who continue to work under coercive conditions rather than at their own discretion and interest can be expected to fail to use effective strategies to solve their multi-role problems and, as a result, face negative psychological and social consequences. Straiton et al. [26] indicated in their qualitative research that working women use maladaptive coping strategies such as avoidance to deal with feelings of guilt over their absence as mothers, work stress, and other pressures from the competitive world of work. The ineffective strategies reported in this study were classified into three subcategories: Fear of risk, a competitive mentality based on win-lose thinking, and failure to take appropriate advantage of opportunities. All three subcategories were in the main category called scarcity mentality. Studies have divided the work vision characteristics of individuals into two categories: Abundance Mentality and Scarcity Mentality. People with scarcity mentality cannot accept or enjoy the success of others [27]. Underlying the scarcity mentality is the belief that competition leads to survival of the fittest. This mindset is closely related to win-lose thinking and influences people's decisions, depriving them of the opportunity to take risks and make progress [27]. Moreover, despite their constant thinking of their own interests, they miss many opportunities for progress in the environment because they do not trust others and see life as if they do not have many options. Studies have shown that people with a scarcity mentality only feel good about themselves when they are better than others [28]. Therefore, it is predictable that these people will always face a series of negative consequences. In this study, participants who used this type of mentality to solve their professional challenges and worries attributed the reason for success to other factors such as luck, connection, rule breaking, etc. Such responses are known in psychoanalysis as defense mechanisms of denial and projection. However, when asked deeper questions, they eventually admitted that they felt envious of successful people or that they were in a kind of competition with them, constantly comparing their shortcomings with those of others. In this study, we observed the effect of another underlying factor called negative personality traits, which plays a role in women's choice of strategies. According to the studies on the concept of scarcity mentality, win-lose thinking leads to fear of failure and rejection, which has a strong negative impact on the quality of relationships while leading to negative perceptions and inadequacies. Therefore, in interpersonal relationships we see intense anger and competition to eliminate the other, and in intrapersonal relationships constant despair, worry, and dissatisfaction with oneself [29]. In the present study, a number of reports identified feelings of jealousy and anger toward others, feelings of inadequacy toward oneself, guilt, and feelings of disinterest toward work. We also witnessed this flawed view of "superiority to housewives" among the participants with a scarcity mentality. Likewise, this finding seems to have arisen under the influence of the unique sociocultural factors, and in some ways, rooted in the notion that these people viewed housework as an insult and a disgrace to themselves.These themes are classified under the category of negative psychological consequences. As shown in the flowchart, it is clear that these negatively perceived consequences can have a reciprocal effect on work performance and the choice of more ineffective strategies. But in addition to negative psychological consequences, narratives of perceptions of negative social consequences were obtained: including two subcategories of perceived gender pressure and pressure resulting from multiple role expectations (Table 2). Indeed, gender is a significant variable in many work-family studies [29, 30], but perceptions of gender pressure for a particular group may be influenced by the characteristics of the context studied. In the present study, some of participants believed that multiple roles, gender characteristics, and the dominant cultural and social context in Iranian families limited their career opportunities and influenced their work experiences. This cultural context creates a sense of failure and leads the Iranian professional women in such a context to spend their entire working lives trying to achieve a leadership position and, when they are forced to give it up or do not get it, they feel defeated and dissatisfied. However, Powell (2018) argued that since the 1970s, gender segregation has declined in most countries due to the increasing employment of women in managerial and professional occupations [31]. According to a social approach, the traits and behaviors of individuals are determined by their gender, and none of the behavioral traits are inherently masculine or feminine, but it is the society and the dominant culture that determine the traits of gender [32]. As we observed in this study, some of the occupations are considered more suitable for women with multiple roles.

Some saw popular grouping as a form of social discrimination and an obstacle to the realisation of their particular dreams. They believed that one of the limitations to getting a job for a woman was gender segregation because it made many environments unsafe and sometimes impossible for a woman to work. According to the participants, a set of unwritten rules based on gender directs women's participation in society, and women who move outside of this direction face intense social pressure. As many studies have shown, preoccupation with gender stereotypes affects people's mental health and impairs their social functioning [33–35].

In explaining the subcategory of the stresses of multi-role expectations, participants acknowledged that working women still play a key role in caregiving, parenting, and household tasks. In a meta-analysis, Shockle et al. (2017) showed that most people view coping with housework as a gendered problem [36]. Recent research shows that working mothers face high maternity norms [37–39], unfair parental leave policies [40], and discrimination in the division of domestic tasks while managing their life tasks and responsibilities within the dominant culture [41]. In Islam and the Iranian legal system, men are expected to pay for family expenses, and women do not have the duty or responsibility to provide for household expenses [42], and even non-working women are not required to do household chores [43–45], but obviously the social roots of patriarchy are stronger than the religious and legal foundations and have permeated the institution of most families.

In some of the narratives of the participants in this study, we heard complaints about the lack of cooperation from the spouse or other family members and the pressure that comes from these expectations. These accounts are consistent with approaches such as role multiplicity, role conflict, and multiple role strain, which suggest that expectations and pressures resulting from multiple concurrent roles threaten the mental health of working women [46]. Limited energy and time constraints can lead to role conflict and role overload, resulting in stress and poor mental health [47]. Egyptian researchers have shown that economic pressures on the family have led Egyptian women to seek employment in recent decades; however, cultural values continue to support traditional roles. Therefore, for Egyptian women, seeking a job and entering a multi-role world leads to negative emotions and fear of neglecting main roles [48]. On the other hand, research has shown that experiencing multiple roles can lower stress levels because women experience organizing themselves to manage multiple roles [49]. Many studies have shown that employment is an important determinant of health and life expectancy [50] [51, 52]. The results of these studies are consistent with the present study on the reported positive social and psychological outcomes and can be explained in accordance with the role expansion hypothesis. This hypothesis states that multiple responsibilities and roles lead to social support, self-esteem, social dignity, and increased mental health through the expansion of the communicationnet work [53]. The positive psychological and social consequences of this study were the result of using effective strategies to solve work problems (Fig. 1). These strategies were divided into two categories: Using an abundance mentality and Having a vision and strategic planning. Abundance mentality leads to taking advantage of opportunities, focusing on one's own abilities, being interested in contributing to collective progress, not being afraid of failure and occupational risks, and thus not suffering from psychological distress, anxiety, and worry caused by comparing oneself to others, blaming oneself, or reflecting on the successes of others [54]. The abundance mentality can help improve a person's performance by reducing their stress and anxiety [55]. The studies and research conducted confirm the above positive effects of an abundance mentality on people's work performance and mental state [56, 57].

The second effective strategy presented in the experiences of the superwomen in this research is determining the career vision and mission and the plan to achieve it (Table 2). The participants who were interested in a job and were free confirmed that they could relax and recover by giving meaning to their career and despite the multi-role challenges. Most of them defined a meaningful mission and goal for their job and tried to achieve it. Studies on the mission of career path and emphasis on the meaning of employment have been conducted since 2007 and have increased in recent years [58]. Examples include the research by Bunderson and Thomson. Their results show that career issuance has a significant negative relationship with high levels of depression [59]. In addition, Duffy and Dik found that career issue has an insignificant relationship with intention to leave the job and a moderate relationship with job commitment, job satisfaction, and organizational commitment [58].

Qualitative research has introduced a five-stage framework for career issuance that ultimately leads to an invaluable work experience [60]. To further explain the category of "effective strategies" and discover its consistency with the pioneering global research, we will explain the two underlying and influential categories in the choice of effective strategies in the work experiences of the superwomen in our research. The participants confirmed that the two categories of "non-cognitive skills" and "personality traits" are among the underlying factors of women's work experiences (Flowchart 1). Following the introduction of the psychological concept of cognitive intelligence in the world of work, the study showed that people do not only enter the labor market with their bodies and minds. They also bring their own unique personal talents [61]. In this study, non-cognitive skills were divided into two subcategories of emotional intelligence and spiritual intelligence. Emotional intelligence is a set of non-cognitive skills that influence an individual's ability to cope with social demands and pressures [62], and it is a key factor in success in school, work, and social settings [63].

Another study concluded that people with high emotional intelligence have higher job satisfaction and organizational commitment and are less likely to suffer from job burnout compared to others [61]. Following the concept of emotional intelligence in the workplace, numerous studies have shown that there are successful organizations today that adapt to different conditions and only organizations that have employees with high spiritual power can act in this way [64]. Spiritual intelligence in the work environment has increased efficiency and productivity [65] and has a positive and significant relationship with physical, mental and social health [66]. Another research reported a significant relationship between employees' spiritual intelligence, spiritual and mental happiness, organizational effectiveness, and increased performance [67]. In line with the findings of the present study, Unterrainer et al. showed in their review paper that spirituality is associated with the enhancement of psychological and personality components [68].

Personality traits are another underlying factor introduced in the present study. Participants argued that superwomen who address their problems and challenges arising from multi-role conflicts with effective strategies and ultimately have a positive and satisfying experience with their careers share a number of personality traits. Participants identified and expressed these traits in themselves or others. The traits most frequently mentioned in the interviews included: seeking novelty and curiosity, humor, optimism and hope, discipline, and perseverance. These results are consistent with the research of Alterman et al. who showed that individuals who score high on the Openness personality trait are curious about the inner and outer world and their lives are rich in inexperience.

In addition, these individuals are less exposed to stress than their counterparts. The results of this study suggest a negative relationship between the Openness personality trait and individual and organizational stress [11]. In explaining the effect of the personality factor of humor, we can point to a more comprehensive personality trait such as extroversion, in which individuals are distinguished based on their interest in others, preference for large groups, courage, activity, conversationalist, cheerfulness, and humor, optimism, determination, and enthusiasm. This outstanding trait makes these people less stressed in the workplace. In addition, such individuals are more likely to have a positive outlook on their future careers and personal competence. This positive cognition and perspective prevent the emergence of negative psychological conditions such as frustration, fatigue and ultimately job stress [12] also, people who have the personality traits of conscientiousness and discipline are accurate, punctual, and reliable, and have high, pre-determined goals and desires. Moreover, people with high scores in this category are conscientious, purposeful, efficient, and determined. It’s been shown that high scores on conscientiousness are associated with job and academic success. Other characteristics of these people are accuracy, punctuality, and striving for success and discipline [69].

## Limitation and suggestion

The present study is limited to describing the experiences of volunteer participants in the cultural context of East Azerbaijan Province. Therefore, the focus of the present study was on a specific context and sample, and such limitations restrict the generalization of the findings to other contexts and ethnic groups. It is recommended that this study be replicated in other contexts and cultures in our country and with other samples. This study provides important themes for experts and researchers to take effective action in providing educational protocols to improve the lives of women with multiple roles by identifying their concerns, challenges, strategies, and the consequences of the choices they face.

## Conclusion

This study is an in-depth investigation of the life experiences of Iranian superwomen, which has also shed light on the paradoxes of these experiences. According to the findings of this study, Iranian superwomen have different work experiences depending on limited work opportunities, the form of educational context and background, compulsory or voluntary employment, personality traits, and non-cognitive skills. Applied strategies of superwomen lead to the perception of different outcomes in solving job challenges, all of which affect the formation of job experiences and ultimately the choice of their next strategies. The present study was conducted knowing the importance of women's role in the three important positions of marriage, motherhood and job. It is worth mentioning that the perception of a super woman's job experience depends on how she plays her multiple roles.

Since there was no in-depth study on the work experiences of Iranian superwomen, we tried to conduct an exploratory qualitative study on the participants' life experiences considering their cultural background. We have not found any other research that accurately addresses the female multi-role community in Iran and other countries and shows the uniqueness of the participants' shared experiences. We also observed a mutual relationship between these categories and tried to show and explain this in the form of a flowchart.

## Supplementary Information

** Additional file 1**. The main interview questions.

## Data Availability

The datasets used or analyzed in the current study are available from the corresponding author upon reasonable request, but the interviews and all coding steps are in Persian and require translation.

## Abbreviations

MHC-SF: mental health continuum-short form
