# Supplementary material for: Iranian superwomen's career experiences: a qualitative study
Source: BMC Womens Health. 2021 May 31;21:227. doi: 10.1186/s12905-021-01369-3 (PMC8166137; doi:10.1186/s12905-021-01369-3)
Supplement: Supplementary file 1 — Additional file 1. The main interview questions. [file 12905_2021_1369_MOESM1_ESM.docx]

**Main question**

"Given having several roles, how do you describe your working life?"

**Sub-questions**

**For example: (**All of this questions maybe not used to all of participants**)**

What factors make this experience harder or easier?

What is the Participants' perceptions of the concept of job for women?

What is the Participants' perceptions of the concept of superwoman?

How to take up job?

What is the reason for continuing the job despite having several other roles?

What is the strategies used to hold balance between personal and work life?

This general questions were evaluated by five experts.
